# SGN-YOLO: Detecting Wood Defects with Improved YOLOv5 Based on Semi-Global Network

**DOI:** 10.3390/s23218705

**Published:** 2023-10-25

**Authors:** Wei Meng, Yilin Yuan

**Affiliations:** 1 College of Information, Beijing Forestry University, Beijing 100083, China; 2 Engineering Research Center for Forestry-Oriented Intelligent Information Processing of National Forestry and Grassland Administration, Beijing 100083, China; echoyuanyl@bjfu.edu.cn

**Keywords:** deep learning, wood defects detection, YOLOv5, attention mechanism, EIOU loss function

## Abstract

Object detection based on wood defects involves using bounding boxes to label defects in the surface image of the wood. This step is crucial before the transformation of wood products. Due to the small size and diverse shape of wood defects, most previous object detection models are unable to filter out critical features effectively. Consequently, they have faced challenges in generating adequate contextual information to detect defects accurately. In this paper, we proposed a YOLOv5 model based on a Semi-Global Network (SGN) to detect wood defects. Unlike previous models, firstly, a lightweight SGN is introduced in the backbone to model the global context, which can improve the accuracy and reduce the complexity of the network at the same time; the backbone is embedded with the Extended Efficient Layer Aggregation Network (E-ELAN), which continuously enhances the learning ability of the network; and finally, the Efficient Intersection and Merger (EIOU) loss is used to solve the problems of slow convergence speed and inaccurate regression results. Experimental results on public wood defect datasets demonstrated that our approach outperformed existing target detection models. The mAP value was 86.4%, a 3.1% improvement over the baseline network model, a 7.1% improvement over SSD, and a 13.6% improvement over Faster R-CNN. These results show the effectiveness of our proposed methodology.

## 1. Introduction

**Figure 1:**
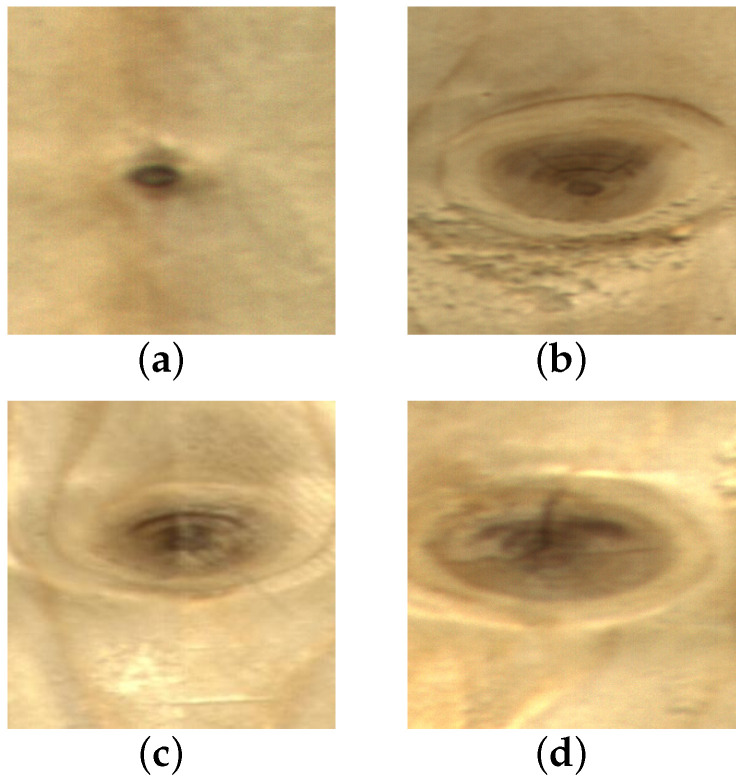
Typical surface defect images of wood. (**a**) live knot; (**b**) live knot; (**c**) live knot; (**d**) knot with crack.

Wood plays a significant role in various industries, such as furniture and construction, due to its naturally renewable, resilient, high strength-to-weight ratio, and lightweight properties. However, its surface inevitably contains defects like live knot, dead knot, resin, knot with crack, and crack [1]. These defects affect wood properties such as toughness and strength during the wood processing stage, significantly reducing the utilization of wood as a raw material [2]. Before further processing, it is necessary to cut off the wood surface defects according to their type and location to maximize the rate of wood yield. Therefore, wood defect detection is essential for the improvement of wood utilization and quality. Some of the defect images are shown in Figure 1. On the one hand, some classes have large intra-class differences; as seen in Figure 1a,b, the scale of the live knots varies greatly. On the other hand, inter-class distinctions are relatively minor. For example, in Figure 1c,d, the difference between a live knot and knot with crack is primarily based on whether a crack exists within the knot’s boundary, which is difficult to recognize by the human eye. In conclusion, the detection of wood defects is rather challenging.

Traditionally, surface inspection has been performed manually. This process is a subjective evaluation and therefore time-consuming and labor-intensive. Research by [3] showed that accuracy rates of 70% were usually difficult to achieve because the human eye was easily fatigued and distracted during the inspection process. Another study indicated that human errors in wood defect inspection result in 22% of waste, reducing the total yield of wood products from 63.5% to 47.4%. As a result, wood was unable to fully realize its potential, and its overall utilization efficiency remains low [4]. Therefore, the detection of wood defects is gradually being replaced by machines and shifting towards automation in order to enhance its comprehensive utilization.

In the early stages, the automation of wood defect detection mainly relied on methods such as stress waves, X-rays, and ultrasonic waves [5,6,7]. The non-destructive assessment of the mechanical properties of wood using stress wave technology is primarily accomplished by measuring the velocity of stress waves. Stress waves propagate in three main forms: axial waves, transverse waves, and surface waves in wood. Among these, axial waves exhibit exceptionally high propagation speeds, and their velocity is closely related to the fundamental transmission speed of wood. However, this equipment demands a relatively high level of environmental control during practical testing, as it necessitates secure attachment to the surface of the wood. The detection of defects in the examined wood by utilizing the X-ray fluorescence imaging effect relies on discerning differences in X-ray intensity after passing through the object under examination. This method effectively distinguishes defects such as dead knots, insect holes, and cracks. The images obtained after irradiating the human body with X-rays tend to exhibit relatively poor quality, resulting in low visual contrast and limited sensitivity in defect identification. This makes it challenging to analyze and identify internal flaws within the wood. The propagation velocity of ultrasonic waves varies in different mediums, and the elastic modulus of wood can be calculated based on the propagation medium. The advantage of ultrasonic technology lies in its ability to reveal the condition of defects both inside and outside the wood. However, ultrasonic waves are susceptible to external influences, which can result in instability.

In recent years, artificial intelligence has gradually emerged and rapidly developed. Machine learning methods that utilize manually selected features have been extensively applied to wood defect detection. Feature extraction is the process of dimensioning an image using mapping or transform projection to reduce redundancy and avoid overfitting. Effective feature extraction can significantly impact the performance of a model. By analyzing the characteristics such as texture, color, and shape within wood defects, feature extraction algorithms select representative subsets of features. This process can effectively enhance the accuracy of subsequent defect detection. Currently, machine learning methods applied to wood defect detection mainly include techniques like Gray Level Co-occurrence Matrix (GLCM), image segmentation, Support Vector Machines (SVM), and wavelet neural network [8]. These detection algorithms provide an automated approach for wood surface defect detection, effectively avoiding the numerous steps of manual and machine detection [9,10]. However, these algorithms require manual extraction of wood defect features, followed by classification and recognition of defects, which can slow down the actual detection speed. Additionally, these algorithms heavily rely on the quality of manually extracted features, leading to poor reusability.

Deep learning techniques can automatically extract image features, which largely reduces the complexity of traditional machine learning algorithms that require manual feature extraction. By integrating detection and classification, it introduces a novel approach to wood defect detection. Researchers have successfully applied Convolutional Neural Networks (CNNs) to wood defect detection and have achieved promising research outcomes. CNNs show remarkable robustness and self-learning capabilities in identifying wood defect patterns. They are not only free from the limitations of human subjectivity and variability, but also eliminate the need for manual feature selection. Currently, two types of CNN architectures are widely used in object detection: two-stage algorithms and one-stage algorithms. Two-stage object detection algorithms mainly include R-CNN, Fast R-CNN, Faster R-CNN, and Mask R-CNN [11,12,13]. Firstly, the candidate regions within the image, followed by the classification and bounding box regression of these candidate regions to obtain the final object detection results. Due to the above two steps, two-stage methods typically require more computational resources and time compared to one-stage detection methods. One-stage detection frameworks mainly consist of the SSD and YOLO series [14,15,16]. They omit the Region Proposal Network (RPN) step and directly obtain classification and local information. In contrast to two-stage methods, one-stage methods are generally easier to implement and deploy. In practical application scenarios, it is important to consider accuracy and efficiency simultaneously. Thus, in this paper, we chose the YOLOv5 detection framework as our baseline. Although YOLO has achieved success in tasks like face detection and pedestrian detection, it still has limitations in wood defect detection scenarios. Due to its difficulty in capturing features from shallow network layers and contextual information, its accuracy noticeably decreases when detecting smaller defects.

To address these challenges, this paper proposed a YOLO-based wood defect detection model utilizing SGN (SGN-YOLO). Furthermore, an enhanced backbone architecture is developed to enhance feature extraction capabilities and overcome the accuracy limitations due to large-scale variations in traditional object detection algorithms. Our main contributions are summarized as follows:

1. We design a semi-global network to replace the C3 module, which focuses on local information and integrates global information simultaneously.
2. We combine the E-ELAN with the depth model to learn more diverse features without disrupting the gradient pathways. Additionally, we improve the loss function by introducing a smoothing term to address the issue of offset and imprecise localization in object detection.
3. We propose a unified framework (SGN-YOLO) for accurate defect detection and conduct extensive experiments on public datasets. Our approach achieved 86.4% in the mean average precision (mAP) metric, surpassing many existing models.

## 2. Related Works

### 2.1. Methods Based on Traditional Machine Learning Algorithms

Wood defect recognition methods based on machine learning typically involve Gray Level Co-occurrence Matrix (GLCM), image segmentation, Support Vector Machine (SVM), and wavelet neural networks.

In order to detect wood surface quality, Wang proposed a method for detecting wood surface quality by extracting gray-scale histogram statistical features and GLCM texture features of the wood [9]. The proposed method utilized more pixel information compared with the traditional four-angle method, thus achieving a higher classification accuracy than the previous method. Hashim performed classification experiments on defects of Meranti timber species based on an orientation-independent GLCM method [10]. The comparison results showed that the proposed feature set had superior classification accuracy. Despite this, GLCM contains all the texture information for the picture and the surplus data, which may result in computer redundancy. Zhang constructed a new defect segmentation methodology using morphological reconstruction with the R component from an RGB image [17]. The method was able to achieve fast and accurate defect segmentation. Chang used convex optimization (CO) as a pretreatment method for smoothing and the Otsu segmentation method to detect wood defects [18]. The performance of segmentation with different CO weights was evaluated by calculating structural similarity (SSIM) results between an original image and a defect image. However, there segmentation of wood defects in practical applications is not sufficient, and it is necessary to classify the types of wood defects. Furthermore, many traditional machine learning methods were used to classify wood defects. In order to identify the internal defect condition of the wood, Gu proposed a tree-structure SVM to detect different knots. The best classification rate was 96.5% [19]. Xu optimized SVM algorithm training by combining genetic algorithm (GA) [20]. Based on the results, GA-SVM was found to produce the best predictions. The SVM classifier is the most common in machine learning, which overcomes the disaster of dimension and non-linear divisibility problems. Nonetheless, in practical application, it could produce a good performance for small samples but not large ones. Zhang proposed a collaborative classification method without image segmentation [21]. By using this method, a 40-dimensional feature vector can be extracted from the plate image by performing a three-level dual-tree complex wavelet decomposition. Experimental results showed that this feature could express complex information about wood surfaces. However, the wavelet decomposition method will produce redundant information, which makes it less readable in the face of objective classification problems with immeasurable information such as defects.

Overall, the above methods are based on a priori wood feature extraction. Due to the different sizes and shapes of the wood surface defects, the texture structure is diverse and complex. Thus, extracting these features is complicated and time-consuming.

### 2.2. Methods Based on Deep Learning Algorithms

With the development of deep learning, many researchers have applied deep learning methods to wood defect detection. Deep learning frameworks can learn wood features automatically without complex calculations. Fan compared several classical algorithms such as R-CNN, SSD, and Faster R-CNN [22]. After optimizing the algorithms, R-CNN achieved breakthroughs in object detection, with improved accuracy compared to other algorithms. However, R-CNN also has limitations, such as the redundancy introduced by overlapping object proposals, thus affecting the speed of object detection. The Faster R-CNN algorithm uses the Region Proposal Network (RPN) to identify wood defects, speeding up defect detection without reducing accuracy. The results showed that Faster R-CNN outperformed SSD in terms of both average detection accuracy and speed. Despite its strong performance, the two-stage nature of the Faster R-CNN algorithm slowed down detection speed, failing to apply to real-time devices. With limited memory and computational resources in the processing platform, it is a challenge for a lightweight device to achieve accurate and fast detection performance. The one-stage detection frameworks mainly include SSD and YOLO series, which omit the process of RPN and obtain the classification and local information directly [14,15,16,23]. Yang used a Deep Residual Network (ResNet) as the backbone of an improved SSD model, replacing the original VGG network [24]. This optimization enhanced the input feature optimization for bounding box regression and classification tasks. The average defect detection accuracy reached 89.7%, with an average detection time of 90 ms. Fang compared YOLOv5, YOLOv3 SPP, and Faster R-CNN on two wood defect datasets [25]. The experimental results showed that YOLOv5 surpassed the other two models in F1-score and showed a significant advantage in training speed. As a result, YOLOv5 was more suitable for the detection of wood surface defects.

## 3. Methodology

### 3.1. Baseline

**Figure 2:**
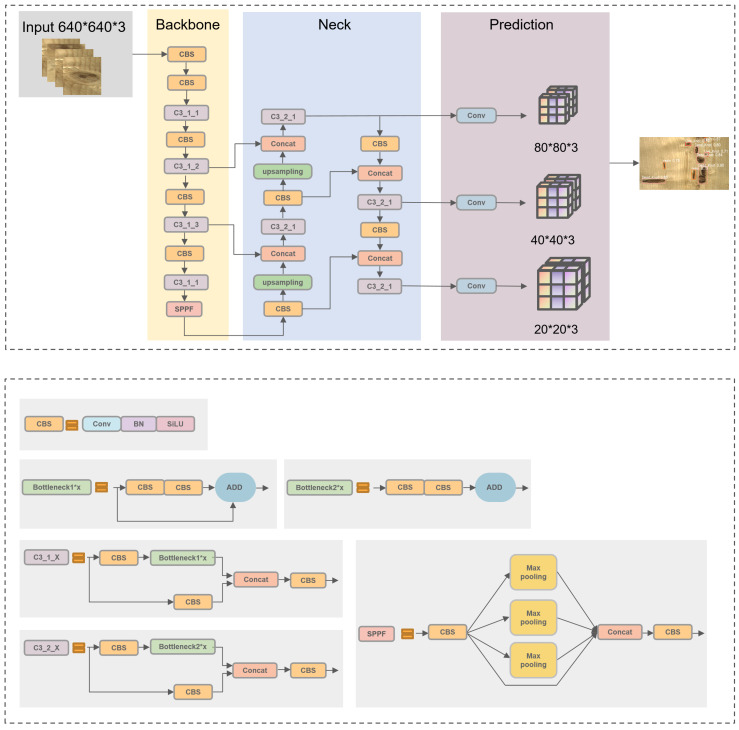
The architecture of original YOLOv5. The network consists of four main parts: input, backbone, neck, and prediction.

YOLOv5 is a one-stage object detection algorithm introduced by Ultralytics in 2020. The model is divided into four parts, and the details of each module are shown in Figure 2.

The input module mainly processes the input images using Mosaic data augmentation. The principle of Mosaic data enhancement is to recombine four different pictures into one picture in a batch size, using random scaling, cropping, and arranging them using CutMix data enhancement [26]. This trick can enrich data diversity, improve the robustness of the network, and reduce costs.

The backbone includes the structure of CBS and SPPF structures. CBS is used for feature extraction in the C3 module, composed of a bottleneck structure and three convolutions. YOLOv5 incorporates two types of C3 structures: C3_1 in the backbone and C3_2 in the neck. SPPF is improved based on SPP, which provides various receptive offer fields to enhance feature representation.

The role of the neck is to improve feature extraction capability. The Feature Pyramid Network (FPN) fuses shallow features with deep semantic information, and the Path Aggregation Network (PAN) merges the extracted features with FPN.

The prediction consists of three prediction layers at different scales to detect large, medium, and small objects, respectively. The final results are obtained by Non-Maximum Suppression (NMS) processing, which helps eliminate redundant boxes. Information from the prediction box with the highest confidence is retained in the final output.

The YOLOv5 series mainly include three models with different weights: YOLOv5s, YOLOv5m, and YOLOv5l. The width and depth increase sequentially, and the speed decreases with increasing parameters. In addition, we introduced two models from the YOLOv7 [27] series: YOLOv7 and YOLOv7-tiny, and compared them with the YOLOv5 series. First, we trained these models separately. The comparison results of the training are shown in Table 1. The mAP of YOLOv5m, YOLOv5l, and YOLOv7 see only a small rise of 3.2%, 3.4% and 3.5%, respectively, compared to YOLOv5s. Nevertheless, the YOLOv5s and YOLOv7-tiny models have fewer parameters and computations than others, making them suitable for embedded devices. In a real-time object detection system, it is not only necessary to consider the accuracy of detection, but also the lightweight of the model. While YOLOv7-tiny exhibits a better recall rate and smaller size, its precision and mAP are 7.2% and 2.1% lower than YOLOv5s, respectively. Therefore, we used the YOLOv5s network in our experiments.

Although YOLOv5 has performed well so far, there remain some shortcomings in detecting small objects and missing detection problems. On the one hand, it is difficult to capture shallow network information due to the neglect of features in the first C3 block; on the other hand, the block is unable to obtain global and contextual information that would increase its efficiency and accuracy of the network. Therefore, we modified its structure by adding SGN and E-ELAN modules.

### 3.2. Semi-Global Network (SGN)

**Figure 3:**
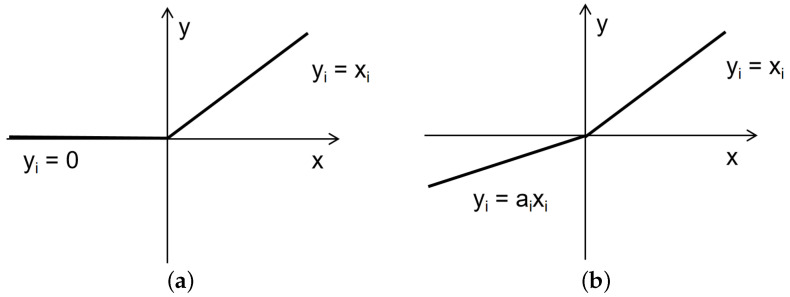
ReLU function and LeakyReLU function. (**a**) ReLU; (**b**) LeakyReLU.

**Figure 4:**
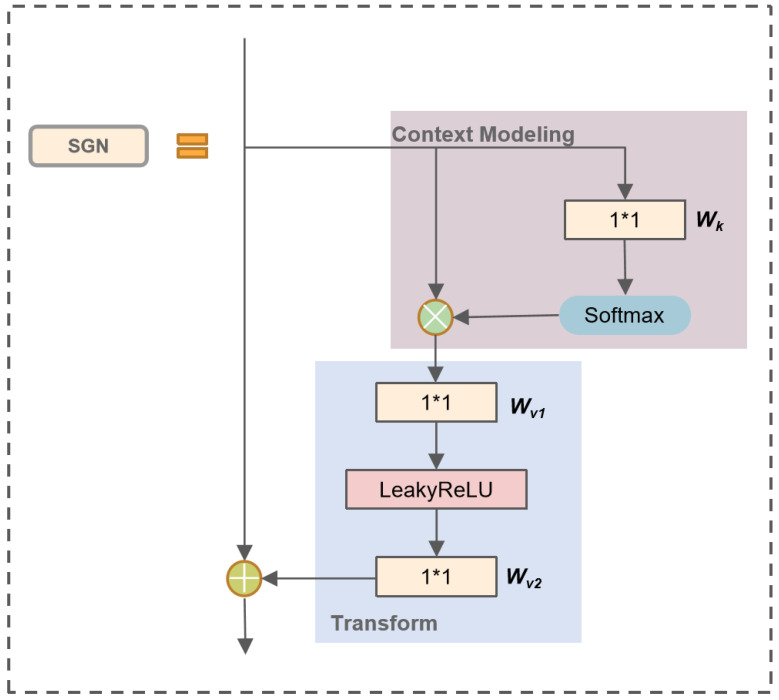
The structure of the SGN.

Recently, various computer vision tasks have been successfully performed using the attention mechanism. To enhance the feature expression ability of YOLOv5, inspired by Global Context Network (GCN) [28], we proposed the Semi-Global Network (SGN) module. In image-related tasks, LayerNorm refers to the normalization of an entire image, which involves calculating the mean and variance across all channels and pixels within the image. SGN eliminates the LayerNorm operation, resulting in faster model inference since it no longer requires the computation of mean and variance. Experimental results indicate that the removal of the LayerNorm operation did not lead to overfitting issues. Additionally, we have replaced ReLU with LeakyReLU. As shown in Figure 3, ReLU turns all negative inputs into zeros, which can easily lead to neuron deactivation. In contrast, LeakyReLU addresses the issue of neuron death by computing gradients for the portion of inputs less than zero during backpropagation, rather than setting them to zero as in ReLU, thus mitigating the sparsity of ReLU. Figure 4 illustrates the structure of SGN. The SGN module consists of global context modeling. The global context or the context information set of the relationship between pixels in the image can help the visual system recognize the spatial layout of the object. For SGN, each region aggregates features from all regions. It is designed to capture multilevel long-range dependencies and global information, and thus enhancing generalization while remaining lightweight. Since the existing methods for integrating context information often require a large amount of computation and memory, more GPU resources are needed for progress. This method adds the same contextual information for all spatial locations to minimize the demand for GPU resources. Due to its lightweight nature, we can apply it to multiple backbone layers. Three SGN modules are embedded into the backbone part of the YOLOv5 network model. The following equation can model the SGN information:

(1) $$y=W_{v1}\sum_{j=1}^{C}\frac{exp(W_{k}x_{j})}{\sum_{m=1}^{C}exp\left(W_{k}x_{m}\right)}x_{j}$$

where $\frac{exp(W_{k}x_{j})}{\sum_{m=1}^{C}exp\left(W_{k}x_{m}\right)}x_{j}$ satisfies the Softmax functional, formulated as

(2) $$Softmax\left(W_{k}x_{j}\right)=\frac{exp(W_{k}x_{j})}{\sum_{m=1}^{C}exp\left(W_{k}x_{m}\right)}$$

The whole module consists of two parts, in the first part, attention weights are derived using a 1*1 convolution $W_{k}$ and Softmax function, and followed by the attention pooling to obtain the global context information. The other part performs feature transform via a 1*1 convolution $W_{v}$, and adopts feature aggregation, where global contextual features are added to each feature at a location. Figure 4 illustrates the detailed structure of the global and local blocks, defined as

(3) $$z_{i}=W_{v2}LeakyReLU\left(y\right)+x_{i}$$

where *i* is the index of query positions. *x* and *z* denote the input and output respectively. We denote $x={\left\{x_{i}\right\}}_{i=1}^{C}$ as the feature map of one input instance, where *C* is the number of positions in the feature map.

### 3.3. Extended Efficient Layer Aggregation Networks (E-ELAN)

Wang proposed E-ELAN based on the foundation of ELAN, which is the basic block for YOLOv7 [27]. As stacking numerous computation blocks can disrupt the stability of a network, the design of E-ELAN aims to enable the network to accommodate more overlapped blocks. This is achieved through minor adjustments to the computation blocks without changing the transition layer. The structure uses the “expand, shuffle, merge” bases to continuously enhance the learning capability of the network without destroying the original gradient path.

**Figure 5:**
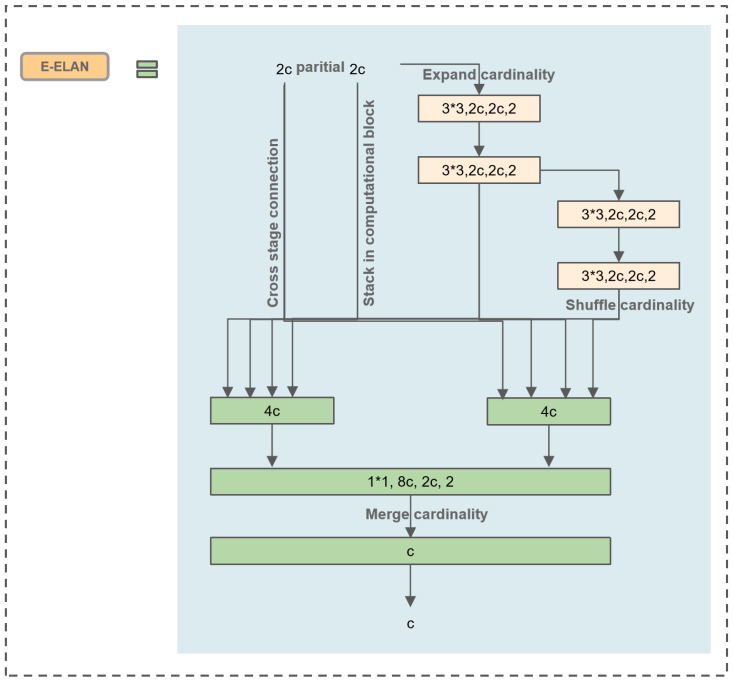
Extended efficient layer aggregation network.

Due to the large model of the YOLOV7 framework, our paper is not based on the complete YOLOv7 framework. Thus, we applied E-ELAN to YOLOv5 to achieve a more efficient network. This approach allows for efficient learning and convergence, making full use of the advantages of YOLOV7. The main structure is depicted in Figure 5, where “c” stands for a convolutional layer, and “4c” stands for four convolutional layers. This modification enables improved network efficiency and performance in the context of wood defect detection.

### 3.4. Efficient Intersection over Union (EIOU) loss

**Figure 6:**
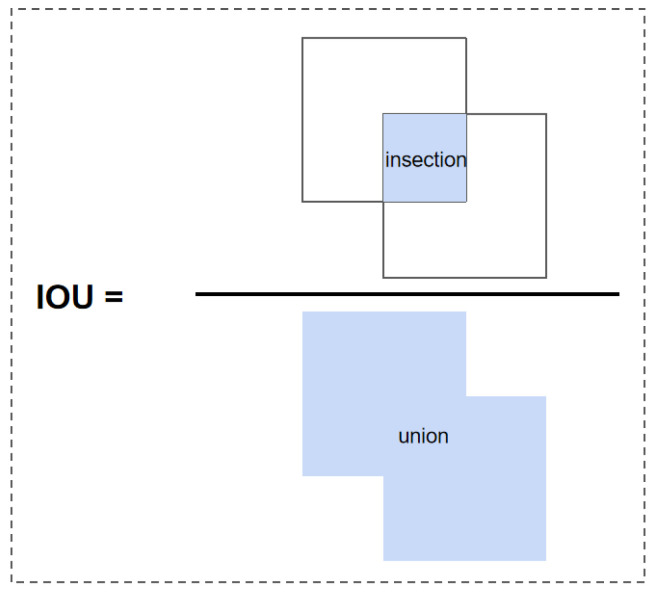
IOU calculation.

The Intersection over Union (IOU) calculates the ratio of the intersection area between the predicted bounding box and the true bounding box to the union area, as shown in Figure 6. The IOU loss function was introduced by face detection technology. It regresses the bounding box formed by the four points of the boundary as a whole, and it takes into account the correlation between the coordinates [29]. However, there are two major disadvantages of IOU loss for bounding box prediction.

**Figure 7:**
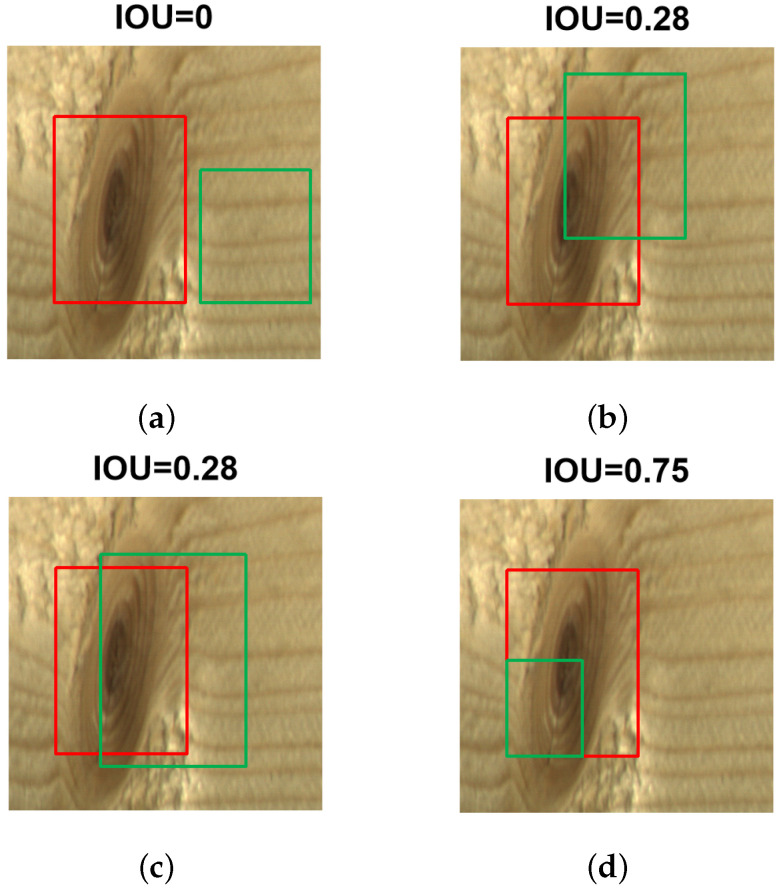
Results of IoU under different intersections. (**a**) IOU = 0; (**b**) IOU = 0.28; (**c**) IOU = 0.28; (**d**) IOU = 0.75.

Firstly, as shown in Figure 7a, the red box represents the true bounding box, and the green box represents the predicted bounding box. When the predicted box and the true box do not intersect at all, the IOU is 0. This can lead to a non-differentiable loss function, making it impossible to optimize when the two boxes do not intersect. Consequently, it becomes challenging to proceed with the next training steps. Furthermore, IOU fails to reflect the degree of intersection. For instance, in Figure 7b,c, the IOU values are the same in both cases. IOU cannot accurately indicate how the two boxes intersect in these scenarios. Rezatofighi proposed the Generalized Intersection over Union (GIOU) to address the above issue by introducing the concept of the minimum enclosing rectangle [30]. From the calculation formula, GIOU not only focuses on the overlapping area, but also on other non-overlapping regions, providing a better reflection of the overlap between the predicted and true boxes. However, when the two boxes are completely overlapped (the predicted box is entirely inside the true box), as shown in Figure 7d, the loss values of GIOU and IOU are the same. In this case, GIOU degrades to IOU and fails to distinguish the true spatial relationship between the predicted and true boxes. This limitation results in slow convergence during the regression process.

Based on this, when the predicted box and the true box do not overlap, the Distance Intersection over Union (DIOU) can minimize the convergence by reducing the distance between the centers of the predicted and true boxes. As a result, DIOU achieves much faster regression convergence compared to GIOU [31]. Further optimization is achieved with the Complete Intersection over Union (CIOU), which builds upon DIOU and considers three important geometric factors: overlap area, center point distance, and aspect ratio. The CIOU boundary box loss function moves the predicted box closer to the true box while ensuring that the aspect ratios of width and height are similar. This speeds up the regression convergence of the predicted box. However, once the widths and heights of the predicted box and true box converge to a linear ratio, it can lead to a situation where the width and height of the predicted box cannot simultaneously increase or decrease during regression.

To solve the above problems, this paper finally adopts the Focal-EIOU approach, which not only considers the overlapping area and center point distance, but also the real differences in width and height the side lengths [32]. Based on CIOU, EIOU solves the ambiguous definition of aspect ratio, and splits the aspect ratio loss item into the difference between the predicted width and height and the width and height of the minimum bounding box, thus accelerating the convergence of the prediction frame and improving the regression accuracy of the prediction frame.

The EIOU loss is calculated by Equation (4), where the loss is computed as an operational function to measure the degree of difference between a predicted box, denoted as *B*, and a target box, denoted as $B^{gt}$. The original YOLOv5 network employed GIOU loss. Compared to the GIOU loss, the EIOU loss achieves faster convergence and superior regression results.

(4) $$\begin{array}{cc} L_{EIOU} & =L_{IOU}+L_{dis}+L_{asp}=1-IOU\left(B,B^{gt}\right)\\ \; & +\frac{\rho^{2}\left(b,b^{gt}\right)}{{\left(w^{c}\right)}^{2}+{\left(h^{c}\right)}^{2}}+\frac{\rho^{2}\left(w,w^{gt}\right)}{{\left(w^{c}\right)}^{2}}+\frac{\rho^{2}\left(h,h^{gt}\right)}{{\left(h^{c}\right)}^{2}} \end{array}$$

where $h^{w}$ and $h^{c}$ are the width and height of the smallest enclosing box covering the two boxes; $w^{gt}$, $h^{gt}$ and *w*, *h* represent the width and height of the real and prediction boxes, respectively. From the Equation (4), EIOU is divided into three parts, $L_{IOU}$, $L_{dis}$, and $L_{asp}$. *b* and $b^{gt}$ denote the central points of *B* and $B^{gt}$, respectively. $\rho \left(\cdot \right)=||b-b^{gt}{\left|\right|}_{2}$ indicates the Euclidean distance; *c* is the diagonal length of the smallest closed box covering both boxes.

## 4. Materials

### 4.1. Experimental Settings and Evaluation Indicators

The experiment was implemented in PyTorch 1.9.0. The network was trained with an NVIDIA GeForce RTX 3090 GPU, and the Compute Unified Device Architecture(CUDA) version is 11.1. All experiments carried out across the different architectures used a standard set of hyperparameters. During the warm-up training prediction, the learning rate was set from 0 to 0.01, and the cosine annealing algorithm was adopted to update the learning rate after the warm-up phase; our model used Stochastic Gradient Descent (SGD) as the optimizer; the batch size was set at 16; the size of the input image was 640*640, and the model was trained for 200 epochs.

### 4.2. Evaluation Indices

We evaluated our approaches using four metrics: Precision (P), Recall (R), Average Precision (AP) and mean Average Precision (mAP), respectively:

(5) $$P=\frac{TP}{TP+FP}$$

(6) $$R=\frac{TP}{TP+FN}$$

(7) $$AP=\int_{0}^{1}P(R)\cdot dR$$

(8) $$mAP=\frac{1}{N}\sum_{i=1}^{N}AP_{i}$$

**Figure 8:**
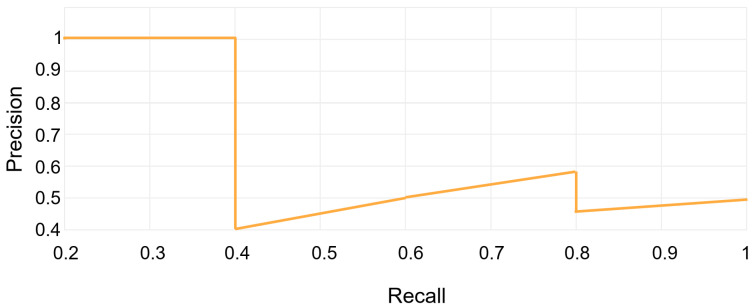
Precision–recall curve.

These metrics are used to assess the quality of the model. TP refers to the number of correct detections made by the model; FP refers to the number of incorrect detections made by the model; FN refers to the number of missed detections made by the model. AP is the weighted average of precision scores obtained at various thresholds along the precision–recall curve. As shown in Figure 8, the general definition of AP involves finding the area under the precision–recall curve above. Precision and recall values always range between 0 and 1. Consequently, AP also falls within the range of 0 to 1. mAP is defined as the average of AP values calculated for all object categories. N is the number of all defect categories.

### 4.3. Image Acquisition

**Figure 9:**
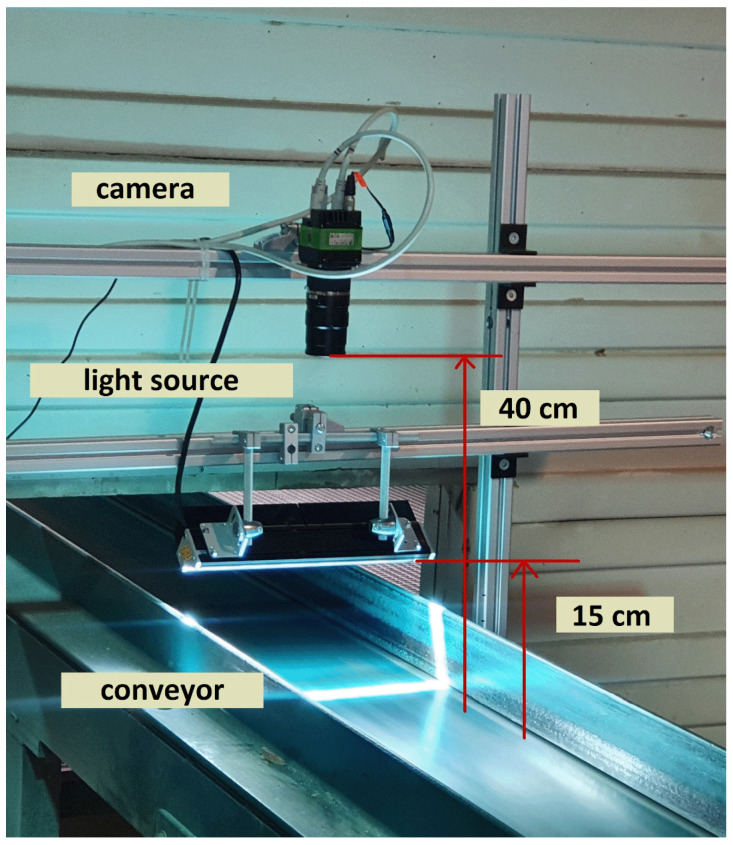
Data acquisition device, including the mounted camera and light source [2].

The dataset was collected from a real industrial environment, including 20,275 original data samples of sawn timber surface and 18,284 images covering ten types of common wood surface defects [2]. As shown in Figure 9, to obtain high-quality images, a portable camera and a mechanical structure with a light source capture images. The resolution for the collected wood was 2800*1024, and a high-performance Camera SW-4000TL-PMCL photographed the images. The original dataset collected was stored in BMP format. Our experiments selected a subset of images from this dataset, including 3910 images, of which 2714 are training, 596 are validation, and 600 are testing. Finally, the Lableme software was used for manual labeling to form the dataset of this research. Five kinds of defects need to be detected: live knot, dead knot, resin, knot with crack, crack, and the probability of defects decreases in descending order. Table 2 summarizes the most common wood surface defects based on their frequency of occurrence.

## 5. Results and Discussion

### 5.1. Comparison Experiment of Different Attention Mechanisms

A noteworthy innovation in the area of computer vision is the attention mechanism. The working principle of the attention mechanism is to make the network adaptable by allocating weight and filtering information [33,34,35]. It enables CNNs to focus more on crucial semantic features and aggregate relevant contextual information with distant dependencies. Without explicit supervision, these mechanisms can learn to emphasize important feature values in a data-driven manner [36]. The Squeeze and Excitation Network (SENet) is an approach that focuses on channel attention, generating attention values for each channel based on global average pooled features [37]. The Convolutional Block Attention Module (CBAM) decomposes the complex attention generation process into different dimensions, reducing overall computational load while enhancing performance [38]. The Global Context Network (GCNet) simplifies non-local mechanisms and inherits the bottleneck structure from SENet, demonstrating superior performance in classification, segmentation, and object detection [28]. Coordinate Attention (CA), introduced by Hou, is an advanced attention model widely employed in object detection [39]. In this work, we integrate the designed SGN attention mechanism into the backbone of YOLOv5 to extract both global and local features.

The original YOLOv5, YOLOv5 + SE, YOLOv5 + CBAM, YOLOv5 + GCN, YOLOv5 + CA, YOLOv5 + GCN and YOLOv5 + SGN were trained, and the performance analysis of these models was listed in Table 3. To compare experimental indicators, the mAP values of YOLOv5, YOLOv5 + SE, YOLOv5 + CBAM, YOLOv5 + GCN, YOLOv5 + CA are 83.3%, 84.9%, 84.7%, 85.4%, and 85.6%, respectively. YOLOv5 + SGN achieves the highest mAP with 86.3%. We can see a 3% rise in the mAP, increasing from 83.3% to 86.3% after the combined SGN attention mechanism.

**Figure 10:**
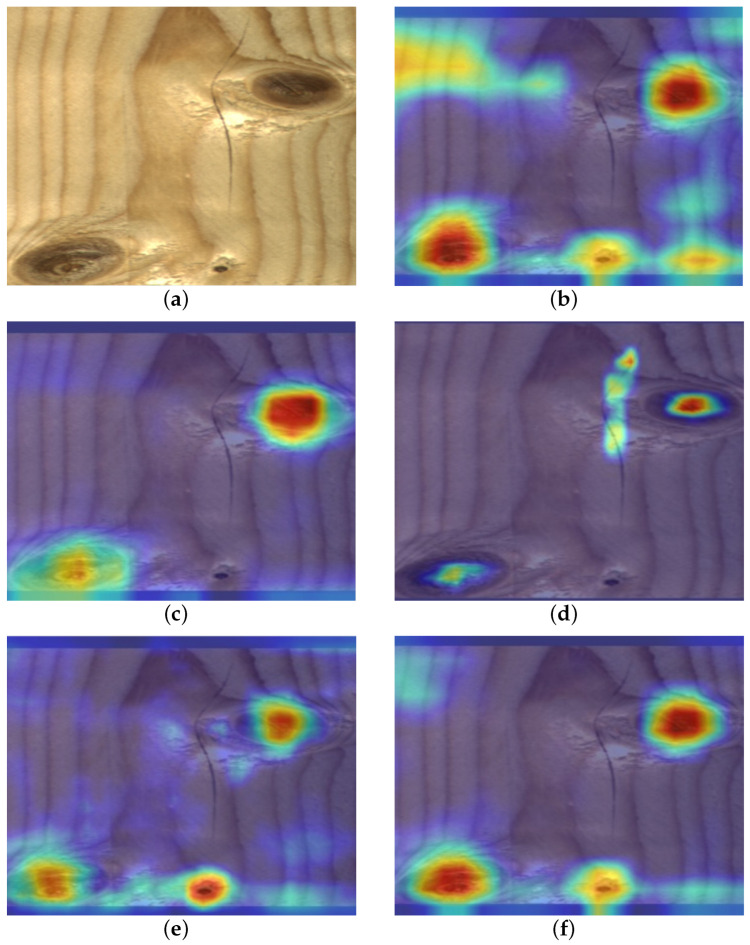
Hotmaps of different attention mechanism modules. (**a**) Original image; (**b**) CA; (**c**) CBAM; (**d**) SE; (**e**) GCN; (**f**) SGN.

Subsequently, we visualized ground truth activation heatmaps using Grad-CAM for the above models, respectively, to explain the effectiveness of the SGN (Figure 10). By observing the generated heatmaps from different models, both SE and CBAM failed to accurately focus on the target region, leading to instances of missed detections. The reason for this phenomenon is that these two forms of attention failed to capture the feature effectively due to the large variation in target scale. From the regions of interest that the model focuses on, the area that the GCN model focuses on is too small to fit the defect boundary well. In contrast, SGN places the heatmap on more important defect areas. For areas that are difficult to distinguish, CA is disturbed by the background, mistaking the natural wood grain for defects. SGN still manages to tightly and relevantly fit the image boundaries, capturing the corresponding targets most accurately. Based on the above analysis, SGN demonstrates superior performance.

**Figure 11:**
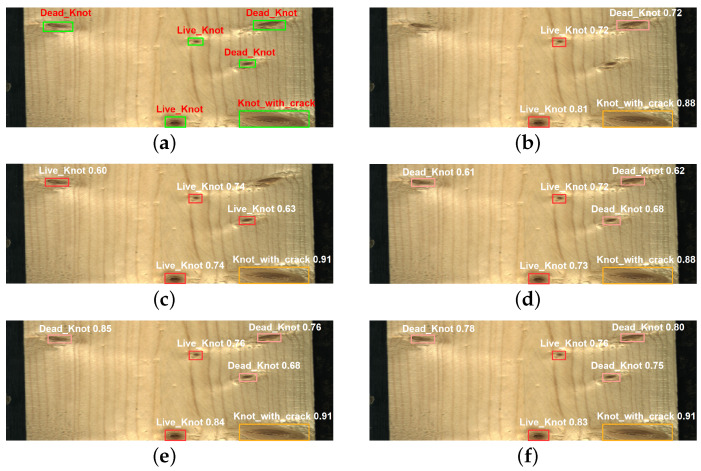
Detection results of five attention mechanisms. (**a**) Manual labeling image; (**b**) YOLOv5 + SE; (**c**) YOLOv5 + CBAM; (**d**) YOLOv5 + GCN; (**e**) YOLOv5 + CA; (**f**) YOLOv5 + SGN.

Additionally, we randomly selected an image to test the detection performance of different attention models, as shown in Figure 11. Both YOLOv5 + SE and YOLOv5 + CBAM models have the same problem of missed detections, and YOLOv5 + CBAM also suffers from a false positive, mistaking a dead knot for a live knot. It is evident that YOLOv5 + SE and YOLOv5 + CBAM have shallower feature extraction and poorer generalization capabilities. Other models do not show any missed or false detections. The YOLOv5 + SGN model successfully identifies all targets while maintaining high confidence scores. Based on these results, it is evident that the SGN attention model is efficient and effective.

### 5.2. Comparison Experiment of Different Loss Functions

**Figure 12:**
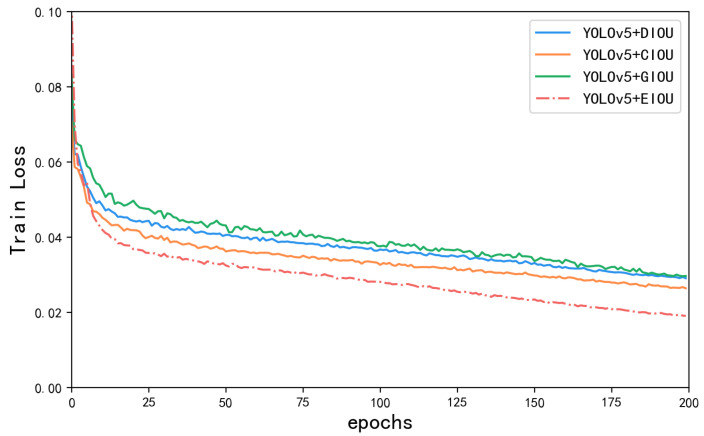
Loss curves of different loss functions.

In object detection, bounding box regression is important for accurate localization. EIOU introduces an additional penalty term to measure the differences in the overlap area, center point, and side lengths in bounding box regression. As a result, it addresses the limitations of existing losses, and balances the gradients from high-quality and low-quality examples [32]. As depicted in Table 4, YOLOv5 achieves the highest accuracy, recall, and mAP performance with the EIOU loss, followed by CIOU and DIOU. The Figure 12 illustrates the loss curves of different loss functions during the training process. From the trend of loss value changes, as the number of training iterations increases, the loss values continuously decrease from an initial value of 0.1%, and finally converges to a lower and stable value. Only the EIOU loss reaches below 0.02%. In terms of convergence speed, EIOU converges faster compared to the other loss functions, while the other models require a longer time to converge. In terms of stability, the final curve shows no obvious oscillations, indicating that the model is more stable in the training process.

### 5.3. Comparison Experiment of Different Algorithms

To validate the performance of the proposed model, we conducted a comparison with state-of-the-art object detection algorithms, including Faster R-CNN, SSD, ResNet + SSD, YOLOv3, YOLOv5 and YOLOv7. The experimental results are shown in Table 5. Compared with other algorithms, we observe that YOLOv7 outperforms other comparable models by 86.8% in terms of mAP. Faster R-CNN shows the highest accuracy in identifying large defects, but its accuracy is lower in identifying small objects. ResNet + SSD achieves the highest precision but has lower recall. SGN-YOLO is more accurate than YOLOv5 itself. Although Faster R-CNN and SSD perform well in certain detection categories, their overall performance is still inferior to SGN-YOLO.

In real industrial scenarios, both the accuracy of detection and the lightweight nature of the model are equally important. They are the main factors to measure whether the proposed model can be applied to industrial scenarios. In terms of frames per second (FPS), the proposed method slightly falls behind ResNet + SSD and YOLOv5. But it can certainly meet the detection requirements of typical surveillance cameras capturing at normal rates of 25 to 30 frames per second [40]. In terms of average detection time, it has good detection speed compared to other models. The mAP of YOLOv7 is slightly higher than that of SGN-YOLO, but its detection speed is far inferior to SGN-YOLO. Based on the analysis of the above results, the SGN-YOLO model is efficient and achieves a good balance between detection speed and accuracy.

**Figure 13:**
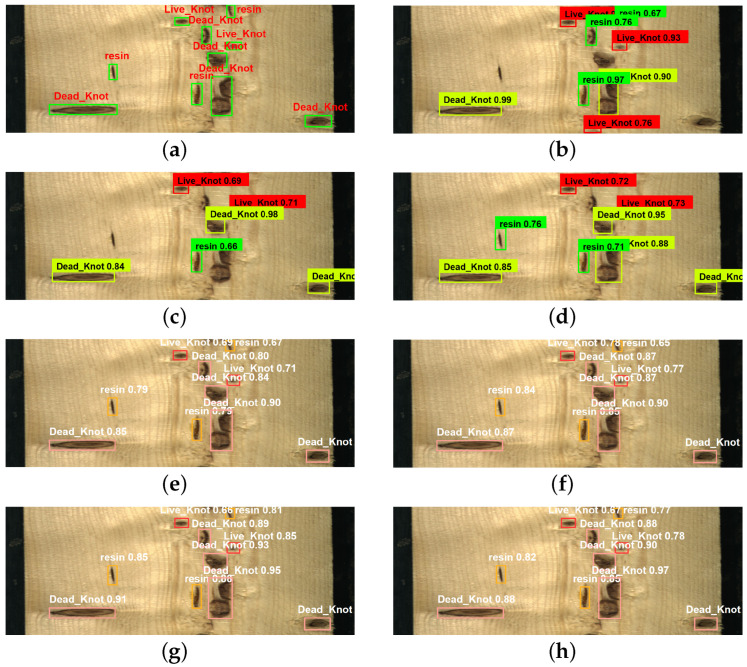
Detection results of five models. (**a**) Manual labeling image; (**b**) faster R-CNN; (**c**) SSD; (**d**) ResNet + SSD; (**e**) YOLOv3; (**f**) YOLOv5; (**g**) YOLOv7; (**h**) SGN-YOLO.

In order to compare the recognition effect of the above algorithms in real industrial scenarios, we selected representative wood images and tested them using different algorithms. From Figure 13, we can see that both Faster R-CNN, SSD, and ResNet + SSD have relatively high confidence scores, but they have different instances of missing detections. Faster R-CNN misses three targets, while SSD misses four targets, which means that these models lose too much information during the downsampling process. ResNet + SSD missed only two targets, indicating that ResNet, compared to the original VGG backbone, can learn more complex feature representations from a deeper network. YOLOv3 and the original YOLOv5 model do not show any missing detections, but the confidence scores of their detection results are not very high. YOLOv7 achieves the highest detection accuracy. However, compared to all other models except for YOLOv7, the improved model exhibits higher confidence in detecting these targets, which ensures the detection of every target and demonstrates superior robustness in complex environments.

### 5.4. Ablation Experiments

This experiment used an ablation study to demonstrate the impact of each improvement module during each installation and removal. As shown in Table 6, after adding the SGN module to the backbone, the total mAP increases by 3%, and the mAP for each defect is also enhanced. Notably, the attention mechanism significantly influences the detection of small defects, such as live knot and dead knot. The replacement loss function EIOU improves the recall from 82.6% to 83.4% and solves the problem of missing targets, with only a slight decrease of 0.4% in mAP. Nevertheless, the introduction of the E-ELAN module raises the mAP metric to 86.4%. With this, we complete the construction of the entire model of SGN-YOLO. The potential reason for the performance drop when adding modules might be the need to find a balance between modules. This ensures optimal performance during the process of adding different modules. Since the order of addition may change the interdependence between modules, different performances can be shown. During our experiment, we tried to adjust the order of module addition, but ultimately found that modeling in the above order resulted in the best performance. In order to ensure the stability and credibility of our final results, we conducted ten repetitions of training for this model and subsequently tested it on the test dataset. The resulting average mAP value was 86.24%, with a standard deviation 0.25, indicating that the data points are relatively close to the mean. Therefore, this is a reasonably stable result. It is worth noting that the difference in mAP values between the test set and the validation set is relatively small, further illustrating the strong generalization capability of our model.

## 6. Conclusions

The detection of wood defects is a major step before wood products are processed. The SGN-YOLO modules were used to detect wood defects, which improved the efficiency and economy of detection. It focuses mainly on the overall design and performance improvement of the network. First, the SGN model was introduced into the backbone to solve the problems of large variations in defect scales and poor detection of small defects. In addition, E-ELAN was introduced to improve the learning ability, which made the network learn more diverse features, and finally, EIOU enhanced the convergence speed in training. Experimental results on an open wood defect dataset showed that SGN-YOLO achieved 86.4% mAP, which is 3.1% higher than the baseline model. The average detection time is 0.015 seconds. We also conducted ablation studies to validate the effectiveness of the improvement modules. Notably, the proposed SGN module contributed the largest accuracy gain (3% increase in mAP, 2.7% increase in precision, and 3.9% increase in recall). Due to its low time consumption and high accuracy, the proposed model can be applied to embedded systems. However, there is still room for improvement in terms of method accuracy. In the future, we aim to reduce detection time further and enhance the accuracy of the algorithm on small defects. Due to the limited availability of open-source wood datasets, validation was performed on a single dataset. In future work, the model will undergo external validation further to enhance the assessment and validation of our model. 

## Figures and Tables

**Table 1:** Comparison of model prediction results.

Models	P (%)	R (%)	mAP (%)	Model Volume (M)
YOLOv5s	83.0	78.7	83.3	14.1
YOLOv5m	83.0	85.7	86.5	42.7
YOLOv5l	83.6	84.1	86.7	91.5
YOLOv7-tiny	75.8	80.4	81.2	12.3
YOLOv7	83.4	85.3	86.8	74.9

**Table 2:** Wood surface defects included in the database with the number of particular occurrences and an overall occurrence within the dataset.

Defect Type	Number of Occurrences	Overall Occurrence in the Dataset (%)
live knot	2981	30.7
dead knot	1846	19.0
resin	2220	22.8
knot with crack	1337	13.7
crack	1319	13.5

**Table 3:** Performance comparison of attention mechanism modules.

Models	Live Knot (%)	Dead Knot (%)	Resin (%)	Knot with Crack (%)	Crack (%)	P (%)	R (%)	mAP (%)
YOLOv5	75.6	80.7	85.4	92.0	82.7	83.0	78.7	83.3
+SE	75.8	88.0	86.9	91.1	82.7	79.9	83.1	84.9
+CBAM	74.0	86.2	90.2	90.1	83.2	80.5	85.5	84.7
+CA	76.5	86.0	89.2	92.9	83.7	81.9	83.5	85.6
+GCN	76.3	87.5	86.8	92.4	83.9	79.8	82.1	85.4
+SGN	76.9	87.4	90.3	92.5	84.4	80.3	82.6	86.3

**Table 4:** Experimental result of different loss functions.

Models	Live Knot (%)	Dead Knot (%)	Resin (%)	Knot with Crack (%)	Crack (%)	P (%)	R (%)	mAP (%)
+GIOU	54.4	59.8	69.5	88.0	75.2	81.7	66.3	71.4
+DIOU	59.3	71.8	71.9	89.1	76.6	76.3	70.4	73.9
+CIOU	61.4	70.3	74.2	90.5	78.2	75.9	71.5	74.9
+EIOU	77.2	87.7	86.8	92.8	84.8	78.1	83.4	85.9

**Table 5:** Experimental comparison of different algorithms.

Models	P(%)	R(%)	mAP (%)	FPS	Average Detection Time (s)
Faster R-CNN	48.0	82.5	72.8	24.0	0.026
SSD	86.5	51.2	79.3	61.2	0.017
ResNet + SSD	87.1	62.3	85.4	68.5	0.015
YOLOv3	80.7	80.7	82.6	42.8	0.028
YOLOv5	83.0	78.7	83.3	52.5	0.016
YOLOv7	83.4	85.3	86.8	54.3	0.030
SGN-YOLO	80.6	82.9	86.4	51.8	0.015

**Table 6:** Ablation experiment comparison.

Baseline	SGN	EIOU	E-ELAN	Live Knot (%)	Dead Knot (%)	Resin (%)	Knot with Crack (%)	Crack (%)	P (%)	R (%)	mAP (%)
YOLOv5				75.6	80.7	85.4	92.0	82.7	83.0	78.7	83.3
+	✓			76.9	87.4	90.3	92.5	84.4	80.3	82.6	86.3
+	✓	✓		77.2	87.7	86.8	92.8	84.8	78.1	83.4	85.9
+	✓	✓	✓	78.0	87.2	90.2	91.3	85.2	80.6	82.9	86.4

## Data Availability

Data will be made available on request.
